# TNF‑*α* Gene Polymorphisms as Determinants of Alloantibody Emergence in Hemophilia: A Systematic Review and Meta‐Analysis

**DOI:** 10.1111/hae.70223

**Published:** 2026-02-07

**Authors:** Alessandra Faustino da Conceição Bezerra, Natã Abner Andrade Carito de Sousa, Suely Meireles Rezende, Renan Pedra de Souza

**Affiliations:** ^1^ Laboratório De Biologia Integrativa Grupo De Pesquisa em Bioestatística e Epidemiologia Molecular Departamento De Genética Ecologia e Evolução Instituto De Ciências Biológicas Universidade Federal de Minas Gerais Belo Horizonte Brazil; ^2^ Departamento De Clínica Médica Faculdade De Medicina Universidade Federal de Minas Gerais Belo Horizonte, Minas Gerais Belo Horizonte Brazil; ^3^ Instituto René Rachou Fundação Oswaldo Cruz Belo Horizonte Brazil

**Keywords:** cytokine, genetic association, immune response, inhibitor development, pro‐inflammatory

## Abstract

**Introduction:**

Inhibitor development remains one of the most serious complications of replacement therapy in patients with hemophilia. Tumour necrosis factor‐alpha (TNF‐*α*) is a key pro‐inflammatory cytokine, and its genetic variants have been implicated in immune‐related conditions. The association between TNF‐*α* gene polymorphisms and inhibitor formation in hemophilia has been explored.

**Aim:**

To systematically review and quantitatively synthesize available evidence on the association between TNF‐*α* gene polymorphisms and the development of inhibitors in patients with hemophilia.

**Methods:**

A comprehensive literature search was conducted in PubMed and SciELO from inception to 11 February 2025. Eligible studies evaluated TNF‐*α* polymorphisms in patients with hemophilia and reported data on inhibitor status. Data extraction and quality assessment (using the Q‐Genie tool) were performed independently by two reviewers. Meta‐analyses were conducted using the Mantel‐Haenszel method, where pooled odds ratios (*ORs*) with 95% confidence intervals (*CIs*) were calculated.

**Results:**

Nineteen studies met the inclusion criteria for the systematic review, and ten were included in the meta‐analysis. A significant association was observed between the rs1800629 (−308*G>A*) polymorphism and inhibitor development under the A‐recessive model (*OR* = 2.00; 95% *CI*: 1.13–3.54). No significant associations were found for other TNF‐*α* polymorphisms.

**Conclusion:**

This meta‐analysis suggests that the TNF‐*α* rs1800629 polymorphism may be associated with an increased risk of inhibitor development in patients with hemophilia. These findings highlight the potential role of inflammatory genetic variants in modulating the immune response to replacement therapy. Further large‐scale, multi‐ethnic studies are needed to confirm these results and better understand the underlying mechanisms.

## 1 Introduction

The immune response against therapeutic factor VIII (FVIII) and factor IX (FIX) in individuals with hemophilia remains a major clinical challenge, particularly due to the development of neutralizing antibodies, also known as inhibitors [1]. Inhibitor formation is triggered by the recognition of infused FVIII or FIX as foreign antigens by antigen‐presenting cells (APCs), leading to T‐cell activation and differentiation, primarily toward a Th1 phenotype. These Th1 cells produce cytokines such as TNF‐*α* and interferon‐gamma (IFN‐*γ*), which promote APC maturation, intensify the immune response, and enhance B‐cell activation, ultimately contributing to the production of anti‐FVIII or anti‐FIX antibodies [2].

Tumour necrosis factor alpha (TNF‐*α*) is a pleiotropic cytokine with both pro‐inflammatory and immunoregulatory functions, produced by various immune cells, including neutrophils, eosinophils, natural killer cells, and activated macrophages [3, 4]. The TNF gene, located on chromosome 6p21.3 within the major histocompatibility complex (MHC) region, is highly polymorphic, and several of its variants have been linked to autoimmune diseases such as celiac disease, Graves' disease, and psoriasis [4, 5, 6, 7]. Among its immunological roles, TNF‐*α* has also been implicated in the pathogenesis of inhibitor development in hemophilia, although its precise role remains unclear [8].

Although the role of TNF‐*α* in inhibitor formation has been investigated at the protein level, the findings have been inconclusive. For example, Santana et al. evaluated serum TNF‐*α* levels in patients with and without inhibitors and found no significant differences, suggesting that circulating levels alone may not capture the complexity of the immunogenetic mechanisms involved in inhibitor development [8].

Another possible explanation of the TNF contribution to the inhibitor development susceptibility is through its genetic diversity. Previous studies have explored associations between TNF‐*α* gene polymorphisms and inhibitor development in hemophilia but due to the small sample sizes typically found in rare disease research, the results have been inconsistent and underpowered. Here, we conducted a systematic review and meta‐analysis aimed to synthesize evidence regarding the potential contribution of TNF‐*α* gene variants to inhibitor formation in hemophilia patients.

## 2 Methods

This systematic review and meta‐analysis were registered in PROSPERO under the registration number [CRD420251057178], and follow the proposed guidelines of the Preferred Reporting Items for Systematic Reviews and Meta‐Analyses (PRISMA) [9].

The literature search was conducted in 11 February 2025, across two biomedical databases (PubMed and Scielo), using a search strategy that combined following terms (“TNF‐alpha” OR “Tumour Necrosis Factor‐alpha” OR “TNF‐*α*” OR “TNFalpha” OR “TNF” OR “tumour necrosis factor”) AND (“hemophilia” OR “haemophilia” OR “hemophilia A” OR “hemophilia B” OR “factor VIII deficiency” OR “factor IX deficiency”). No restrictions were applied regarding publication year or language.

Studies were included if they evaluated the association between TNF‐*α* polymorphisms and the presence of inhibitors in individuals with hemophilia. Studies were excluded if they did not meet the inclusion criteria, or if they were review articles, case reports, or based on animal models.

Data extraction and quality assessment were performed independently by two reviewers, with disagreements resolved by a third reviewer. The methodological quality of the included studies was assessed using the Q‐Genie tool. It evaluates 11 criteria related to study design, genotyping, statistical analysis, and interpretation. Each item is rated on a 7‐point scale, and studies are classified as poor, moderate, or good quality based on the total score. Meta‐analyses were performed under a random‐effects model due to expected heterogeneity in study populations and methodologies.

The meta‐analysis was conducted using R software (version 4.3.3), employing the *metabin* function from the meta package. Pooled odds ratios (*ORs*) and 95% confidence intervals (*CIs*) were calculated to estimate the association between TNF‐*α* polymorphisms and the presence of inhibitors in individuals with hemophilia. Statistical heterogeneity was assessed using the *I* ^2^ statistic and the Chi‐squared test, with *I* ^2^ values above 50% indicating substantial heterogeneity. Potential publication bias for the main significant finding was assessed by visual inspection of the funnel plot and formally evaluated using Egger's linear regression test of funnel plot asymmetry. Finally, to address potential heterogeneity related to hemophilia type, a sensitivity analysis was conducted for the main finding by recalculating the meta‐analysis after excluding studies that focused on Hemophilia B.

## 3 Results

**FIGURE 1:**
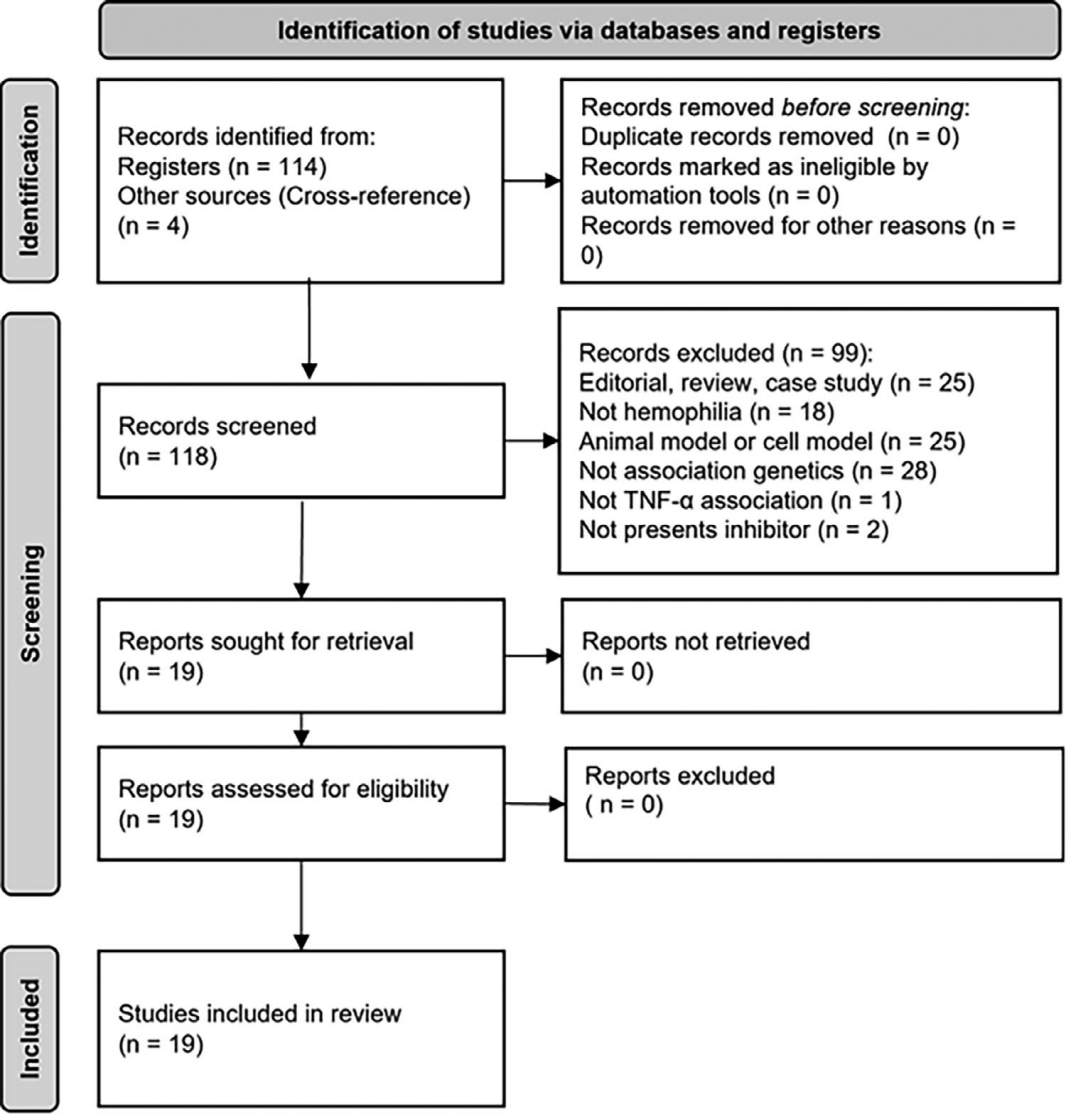
PRISMA 2020 flow diagram showing the selection process of studies included in the systematic review and meta‐analysis.

A total of 114 articles were identified in the databases and were screened (Figure 1). A total of 19 studies met the inclusion criteria and were included in the qualitative synthesis [10, 11, 12, 13, 14, 15, 16, 17, 18, 19, 20, 21, 22, 23, 24, 25, 26, 27, 28], of which 10 were eligible for quantitative analysis [12, 13, 15, 17, 19, 20, 23, 26, 27, 28]. The nine studies included in the qualitative synthesis but excluded from the quantitative analysis did not report sufficient extractable data (such as genotype frequencies in the inhibitor and non‐inhibitor groups) required for statistical pooling in the meta‐analysis, even though they met the general inclusion criteria for the review. The selected studies were published between 2006 and 2021 and involved a combined total of 3538 individuals with both types of hemophilia (Table 1). The TNF‐*α* polymorphisms most frequently investigated were −857C>*T* (rs1799724), −308G>*A* (rs1800629), −238G>*A* (rs361525) and 670 *A>G* (rs3093662).

**TABLE 1:** Summary of the characteristics of the studies included in the review.

Study	Country	Sample size	Inhibitor positive	Type of hemophilia	HA severity	Q‐Genie score^*^
Susanah et al. 2021	Indonesia	216	90	A	Severe	—
Ulrich‐Merzenich et al. 2019	Germany	237	119	A	—	—
Bachelet et al. 2019	Germany	142	62	A	Severe	36.8
Sooriet al. 2019	Iran	39	19	A	Severe	43.5
Chuansumrit et al. 2017	Thailand	136	45	A and B	Severe, moderate and mild	—
Marchione et al. 2017	Argentine	222	91	A	Severe	35.5
de Alencar et al. 2015	Brazil	117	35	A	Severe	—
Zhou et al. 2015	China	52	11	B	Severe and moderate	42.8
Pergantou et al. 2013	Greece	52	28	A	Severe	—
Agostini et al. 2012	Brazil	136	39	A	Severe	30.5
Pinto et al. 2012	India	120	50	A	Severe	37.8
Wieland et al. 2011	Germany	15	4	B	Severe and moderate	—
Lu et al. 2012	China	122	63	A	Severe and moderate	36.5
Zhang et al. 2011	China	140	34	A	Severe, moderate and mild	43.7
Lozier et al. 2011	Multicentric (North America and Europe)	935	302	A	Severe	—
Chaves et al. 2010	Brazil	60	30	A	—	—
Bafunno et al. 2010	Italy	373	113	A	—	47
Pavlova et al. 2009	Germany	260	130	A	Severe	48.5
Astermark et al. 2006	Multicentric	164	63	A	Severe, moderate and mild	45.7

* Q‐Genie quality assessment was applied only to studies included in the meta‐analysis.

The quality appraisal using the Q‐Genie tool revealed that the majority of the included studies were of moderate to high methodological quality. Specifically, three studies were rated as high quality, seven as moderate, and one as poor quality. The overall mean score was 39.73 (*SD* = 1.36), indicating a generally reliable body of evidence.

**FIGURE 2:**
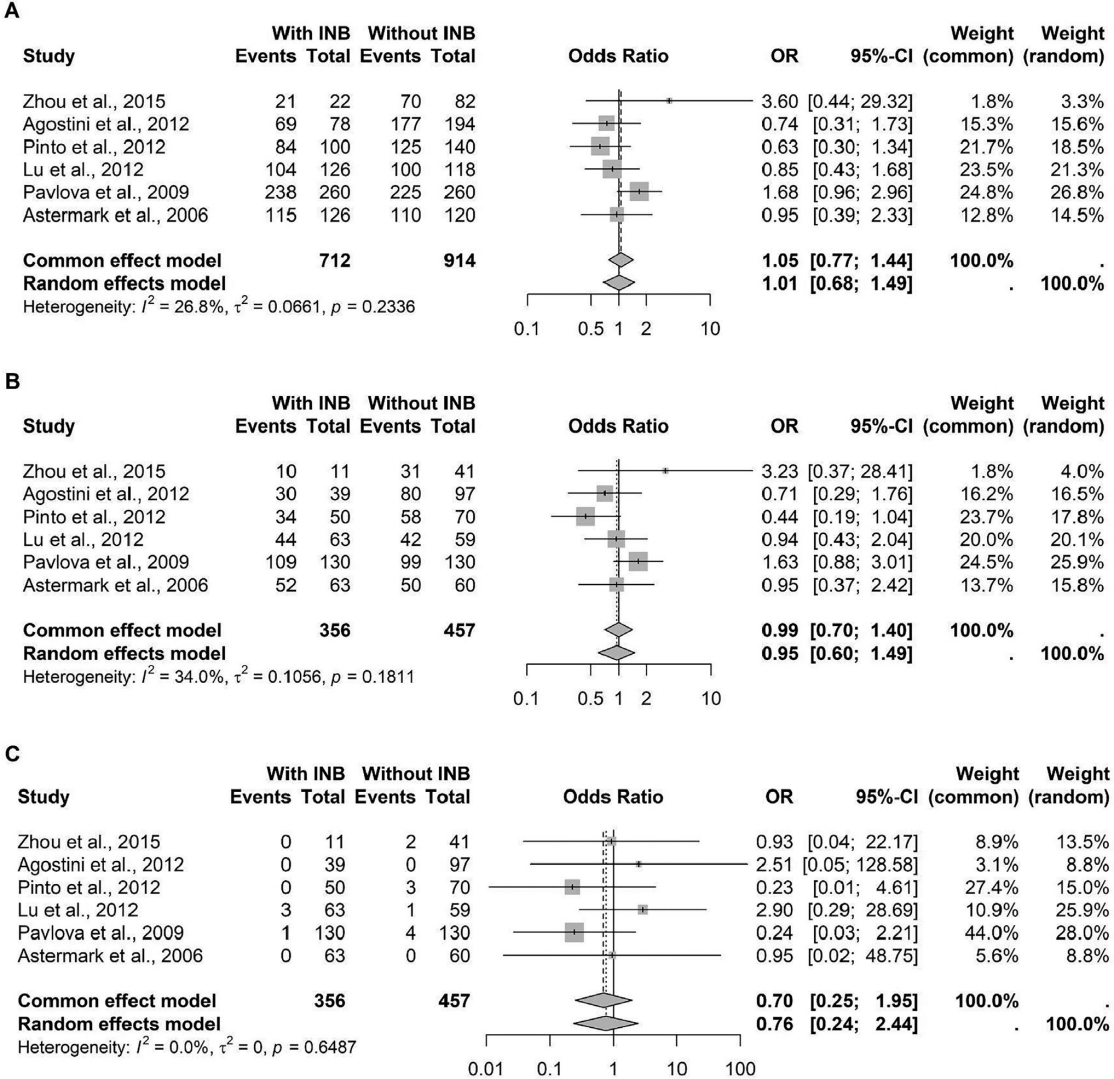
Forest plot illustrating the association of TNF‐*α* rs1799724 in patients with hemophilia, with and without inhibitors. (A) Association with the C allele. (B) C recessive model. (C) T recessive model.

The meta‐analysis was conducted separately for each TNF‐*α* polymorphism. For the rs1799724 variant, a cytosine‐to‐thymine transition at the position‐857, data from six studies comprising 1626 patients were pooled. Under the C‐allelic model, no statistically significant association was found with inhibitor development. Similar results were observed for the C‐recessive and T‐recessive models (Figure 2).

**FIGURE 3:**
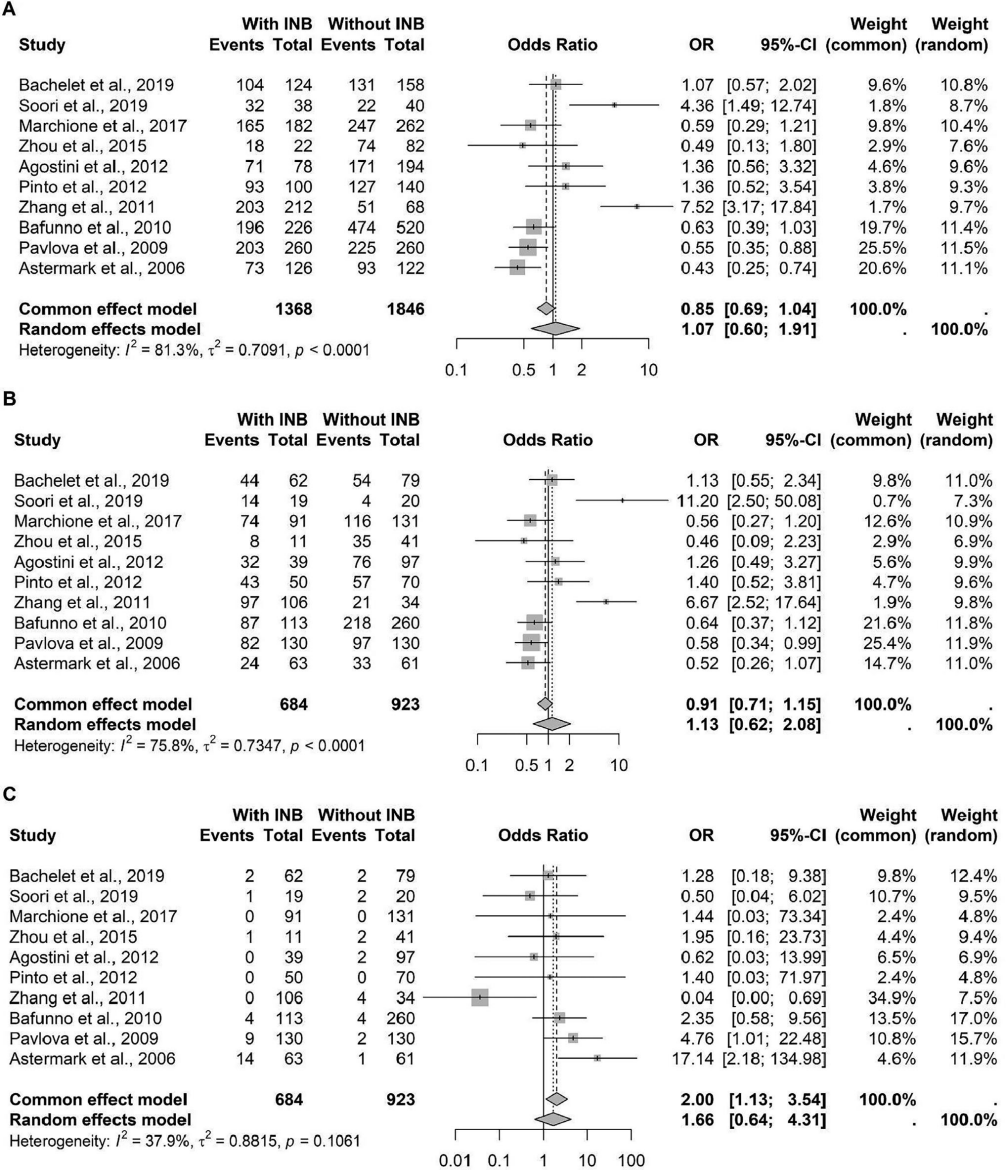
Forest plot illustrating the association of TNF‐*α* rs1800629 in patients with hemophilia, with and without inhibitors. (A) Association with the G allele. (B) G recessive model. (C) A recessive model.

For the rs1800629 polymorphism, a guanine‐to‐adenine transition at the position −308, data from 10 studies comprising 3214 patients were pooled. Under the G‐allelic model, no significant association was found, as well as in the G‐recessive model. A significant association was found in the A‐recessive model, with a common‐effect model showing an *OR* of 2.00 (95% *CI*: 1.13–3.54) (Figure 3). To determine if this association was influenced by the inclusion of the single Hemophilia B study, we performed a sensitivity analysis. After removing the study by Zhou et al. (2015), the association in the remaining nine Hemophilia A studies remained robust and significant (*OR* = 2.00, 95% *CI*: 1.11–3.60) (Data not shown).

**FIGURE 4:**
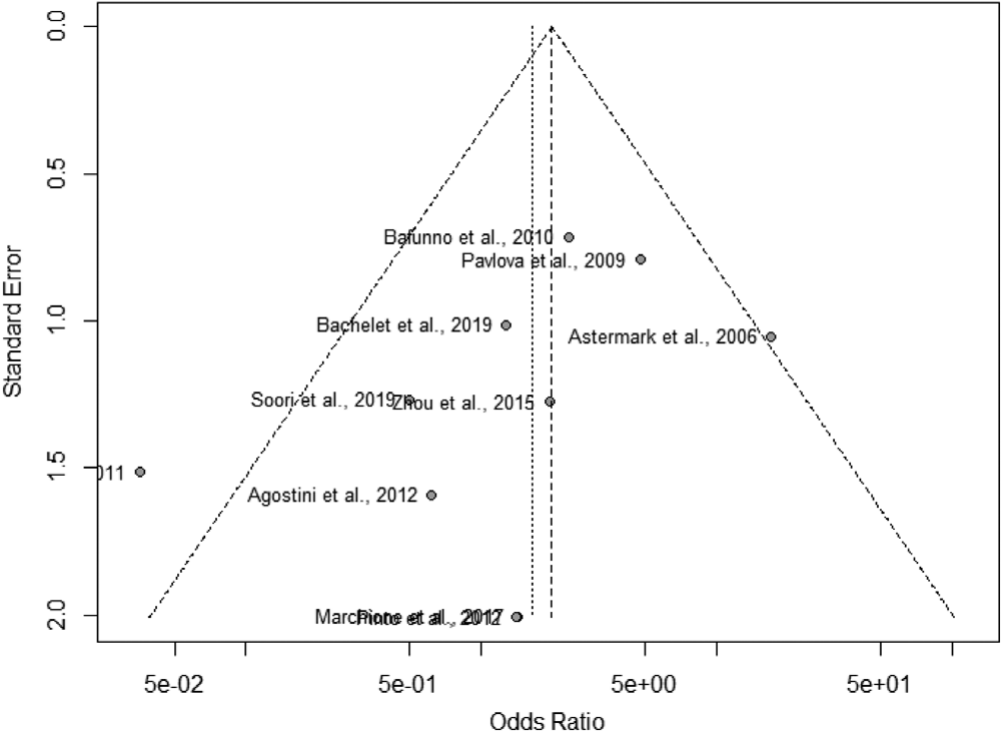
Funnel plot for the *TNF‐α* rs1800629 A‐recessive model. The plot shows the study‐specific ORs (*x*‐axis) against their standard error (*y*‐axis). The vertical dashed line represents the pooled odds ratio. Egger's regression test showed no statistically significant evidence of publication bias (*p* = 0.1628).

We assessed potential publication bias for the rs1800629 A‐recessive model. Although visual inspection of the funnel plot suggested some potential asymmetry, the formal Egger's regression test showed no statistically significant evidence of publication bias (*t* = −1.54, *p* = 0.1628) (Figure 4).

**FIGURE 5:**
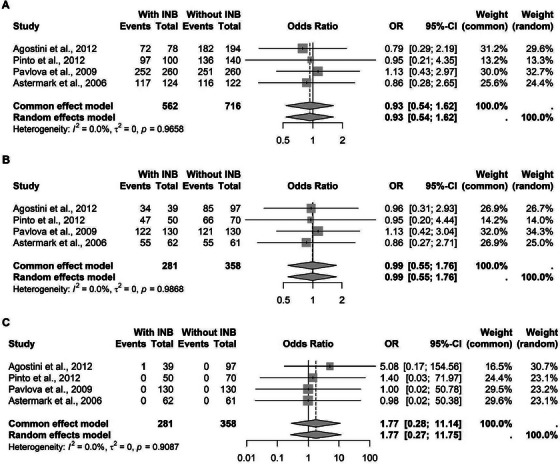
Forest plot illustrating the association of TNF‐*α* rs361525 in patients with hemophilia, with and without inhibitors. (A) Association with the G allele. (B) G recessive model. (C) A recessive model.

For the rs361525 polymorphism a guanine‐to‐adenine transition at the position −238, four studies met the criteria for inclusion, and the pooled analysis did not reveal a significant effect on inhibitor formation (Figure 5).

**FIGURE 6:**
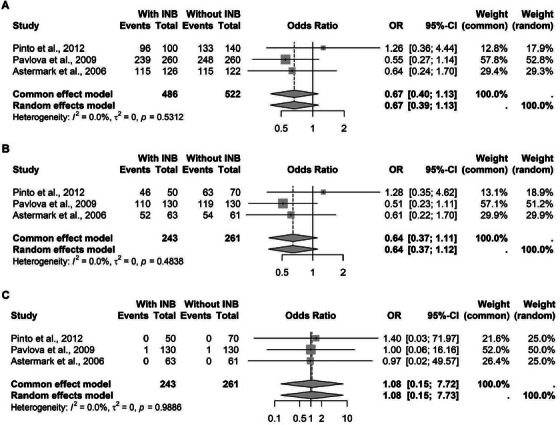
Forest plot illustrating the association of TNF‐*α* rs3093662 in patients with hemophilia, with and without inhibitors. (A) Association with the A allele. (B) A recessive model. (C) G recessive model.

Regarding the rs3093662 polymorphism, an adenine‐to‐guanine transition at the position 670, only three studies comprising 1008 patients were pooled, and likewise, no significant association was identified across genetic models (Figure 6).

## 4 Discussion

This systematic review and meta‑analysis evaluated whether TNF‑*α* polymorphisms influence the emergence of neutralising antibodies in haemophilia. Ten eligible studies were pooled, and only the rs1800629 variant (−308 *G>A*) showed a significant association: carriers of the *AA*/*GA* genotypes had roughly twice the odds of developing inhibitors compared with *GG* homozygotes (*OR*  =  2.00; 95 % *CI* =  1.13–3.54). No convincing evidence emerged for any other TNF‑*α* single‑nucleotide polymorphism.

Functionally, the rs1800629 A allele up‑regulates TNF‑*α* transcription, leading to higher circulating cytokine concentrations in several inflammatory and autoimmune conditions [5, 29, 30, 31]. Elevated TNF‑α levels can promote maturation of antigen‑presenting cells and skew T‑helper responses toward a pro‑inflammatory Th1 profile, both processes that may lower the threshold for breaking tolerance to infused FVIII or FIX and thus facilitate inhibitor formation. By contrast, the remaining variants analysed either lack demonstrated effects on TNF‑*α* expression or were studied in cohorts too small to detect modest genetic influences. Moreover, inhibitor risk arises from a complex interplay between host genetics, treatment intensity, immune status at first exposure and HLA background, which may dilute weak single‑locus effects when studies are underpowered [32].

Several caveats temper these findings. First, the evidence base is limited: each polymorphism was examined in relatively few, mostly small cohorts, reflecting the rarity of haemophilia and the logistical challenges of genetic research in this field. Second, most investigations provided scant information on ethnicity, baseline disease severity or treatment regimen, precluding stratified analyses that could unmask population‑specific effects or gene‐environment interactions. Third, inconsistency in genotyping methods and outcome definitions introduced residual heterogeneity that could not be fully resolved by meta‑analytic models. Although Egger's test did not detect significant publication bias for our main finding (*p* = 0.1628), we acknowledge that the power of this test is limited by the number of included studies (*k* = 10).

Furthermore, given the TNF gene's location within the MHC region, a relevant caveat is the potential for linkage disequilibrium between the rs1800629 variant and specific HLA alleles. This could confound the functional interpretation of our findings, as HLA types are known risk factors. Future studies should ideally co‐genotype TNF‐*α* and HLA variants to dissect their independent or joint contributions to inhibitor risk.

In summary, current data implicate the TNF‑*α* rs1800629 variant as a putative genetic determinant of inhibitor development, whereas other TNF‑*α* polymorphisms remain unsubstantiated. If validated, this variant could eventually be incorporated into genetic risk panels to help stratify patients at higher risk for inhibitor development before initiating therapy. Larger, multi‑centre and consortium studies that combine rigorous phenotyping with uniform genotyping and adequate control for clinical covariates will be essential to confirm this association and to clarify how pro‑inflammatory cytokine pathways intersect with immune tolerance to clotting‑factor replacement.

## Funding

This study was funded by the Coordenação de Aperfeiçoamento de Pessoal de Nível Superior ‐ Brasil (CAPES), Fundação de Amparo à Pesquisa do Estado de Minas Gerais (FAPEMIG), and Conselho Nacional de Desenvolvimento Científico e Tecnológico (CNPq).

## Ethics Statement

Ethical approval was not required for this study, as it is a systematic review based exclusively on previously published data.

## Data Availability

All data analyse in this systematic review are derived from published studies and publicly available sources. No new datasets were generated or analysed for this study.
